# Sourcing cells for *in vitro* models of human vascular barriers of inflammation

**DOI:** 10.3389/fmedt.2022.979768

**Published:** 2022-10-21

**Authors:** Molly C. McCloskey, Victor Z. Zhang, S. Danial Ahmad, Samuel Walker, Samantha S. Romanick, Hani A. Awad, James L. McGrath

**Affiliations:** ^1^ Department of Biomedical Engineering, University of Rochester, Rochester, NY, United States; ^2^ Center for Musculoskeletal Research, University of Rochester Medical Center, Rochester, NY, United States; ^3^ Department of Orthopaedics, University of Rochester Medical Center, Rochester, NY, United States

**Keywords:** vascular heterogeneity, transmigration, endothelial cell, pericyte, inflammation, tissue chip, microphysiological system, organ-on-chip

## Abstract

The vascular system plays a critical role in the progression and resolution of inflammation. The contributions of the vascular endothelium to these processes, however, vary with tissue and disease state. Recently, tissue chip models have emerged as promising tools to understand human disease and for the development of personalized medicine approaches. Inclusion of a vascular component within these platforms is critical for properly evaluating most diseases, but many models to date use “generic” endothelial cells, which can preclude the identification of biomedically meaningful pathways and mechanisms. As the knowledge of vascular heterogeneity and immune cell trafficking throughout the body advances, tissue chip models should also advance to incorporate tissue-specific cells where possible. Here, we discuss the known heterogeneity of leukocyte trafficking in vascular beds of some commonly modeled tissues. We comment on the availability of different tissue-specific cell sources for endothelial cells and pericytes, with a focus on stem cell sources for the full realization of personalized medicine. We discuss sources available for the immune cells needed to model inflammatory processes and the findings of tissue chip models that have used the cells to studying transmigration.

## Introduction

Infection triggers a series of events within a tissue, including tissue-resident immune cell polarization, endothelial activation, and immune cell recruitment. The endothelium plays a central role in this response by modulating blood flow, initiating the coagulation cascade, and assisting in immune cell transendothelial migration (TEM) from the blood into the infected tissue. Although this cascade is largely conserved, endothelial cells (ECs), and their underlying support cells, throughout the body have nuanced differences that enable them to best serve and protect the underlying tissue from disease. During the past few decades, great progress has been made in understanding this tissue-specificity of the vasculature, and within just the last few years several reviews have been published to help researchers apply this knowledge to *in vitro* model systems (1–6). In this review, we will briefly cover the most up-to-date knowledge of vascular heterogeneity of several key organs and discuss how we can reasonably apply this knowledge to human *in vitro* models in the context of immune cell migration. We will highlight key cell types that comprise the vasculature and immune system, covering possible cell sources, including stem cell differentiation protocols. As TEM occurs mostly within microvasculature, this will be our focus of discussion for EC heterogeneity and organ models.

The cardiovascular system is the earliest system to arise during embryonic development. As other organ systems form, they require oxygen and nutrients supplied by this early vascular network (7, 8). The process of *de novo* vessel formation, termed vasculogenesis, is initiated by formation of the vascular plexus from vascular progenitor cells that are derived from the mesoderm, known as angioblasts. Following EphrinB2/EphB4-mediated cell repulsion, angioblasts separate into arterial and venous territories and further differentiate into arterial and venous phenotypes (9, 10). This differentiation was initially thought to be flow driven, but several studies have conclusively demonstrated that these phenotypes arise prior to the heartbeat (9). Rather, nerve-derived signals promote arterial differentiation and alignment of blood vessels, and the cells are then further matured by exposure to higher blood pressure and flow. One key mediator in arterial *vs.* venous specification is vascular endothelial growth factor (VEGF). High levels of VEGF contribute to arterial specification, and lower levels to venous specification (11). From these initial blood vessels, the process of angiogenesis begins, in which new blood vessels branch from the existing ones. The sprouting vessels find each other, forming capillary networks which are reiteratively formed, remodeled, and pruned. For more detail on vasculogenesis and angiogenesis, we recommend a set of excellent reviews (9, 10, 12). Importantly, as the vasculature develops and matures, it recruits a variety of support cells, including pericytes (PC) and smooth muscle cells (SMC), for stabilization. Finally, as organs and tissues develop, the endothelium specializes itself to best serve the organ it vascularizes. Some EC specificity is mitotically stable, whereas other phenotypes arise from outside cues and are lost upon removal from the microenvironment (13–17).

**Figure 1:**
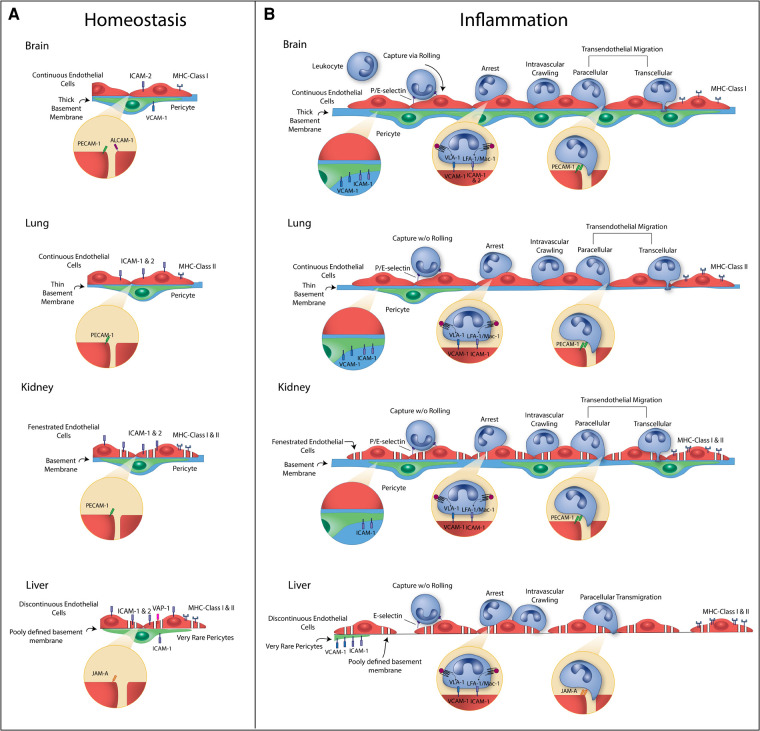
Summary of vascular heterogeneity during homeostasis (**A**) and inflammation (**B**). Heterogeneity illustrated includes endothelial cell (EC) type, EC to pericyte (PC) ratios, basement membrane (BM) characteristics, and leukocyte adhesion molecule (LAM) and major histocompatibility complex (MHC) expression. **Brain:** Brain microvessels have continuous ECs with a high EC : PC ratio and thick basement membrane. During homeostasis, there is very low expression of LAMs and MHC molecules. E-selectin and P-selectin are upregulated during inflammation and contribute to leukocyte rolling. ICAM-1, ICAM-2, and VCAM-1 are upregulated and contribute to leukocyte arrest and crawling. PECAM-1 at cell junctions assists in leukocyte transmigration (TEM), and ICAM-1 and VCAM-1 are upregulated on PCs to guide leukocytes through the BM into the tissue. MHC Class I molecules can be upregulated, which signal to T cells. **Lung***:* Lung ECs at the air-blood barrier are continuous. There is a relatively low EC:PC ratio and thin basement membrane. During homeostasis, there is low expression of LAMs and MHC molecules. E-selectin and P-selectin can be upregulated during inflammation but do not contribute leukocyte rolling. ICAM-1 is upregulated and contributes to leukocyte sequestration and arrest. PECAM-1 at cell junctions assists in leukocyte TEM, and ICAM-1 and VCAM-1 are upregulated on PCs. MHC Class II molecules can be upregulated, which signal to T cells. **Kidney:** Kidney glomerulus capillaries have fenestrated ECs with a moderate EC:PC ratio and thick basement membrane. During homeostasis, there is constitutive expression of ICAM-1 and ICAM-2 and low expression of MHC molecules. E-selectin can be upregulated during inflammation, but leukocyte rolling is debated in the glomerulus. Glomerular ECs rely on platelets for P-selectin. ICAM-1 and VCAM-1 are upregulated and contribute to leukocyte arrest and crawling. PECAM-1 at cell junctions assists in leukocyte TEM, and ICAM-1 is upregulated on PCs to guide leukocytes through the BM into the tissue. MHC Class I and II molecules can be upregulated. **Liver:** Liver sinusoidal capillaries have discontinuous ECs with a low EC:PC ratio and poorly defined basement membrane. During homeostasis, there is expression of ICAM-1, ICAM-2, VAP-1 and MHC molecules. E-selectin can be upregulated in a subset of ECs during inflammation but do not contribute leukocyte rolling. ICAM-1, VCAM-1, and VAP-1 are upregulated and contribute to leukocyte arrest and crawling. JAM-A at cell junctions assists in leukocyte TEM, and ICAM-1 and VCAM-1 are upregulated on PCs. MHC Class II molecules can be upregulated.

While the mature vasculature was initially thought to serve as a passive barrier whose main function was to supply oxygen and nutrients to tissues, a host of studies over the last several decades have elucidated multiple roles of a very active endothelium. These roles are discussed in several excellent book chapters and review articles (7, 18–21). In healthy conditions the vasculature is largely quiescent; however, research suggests ECs play a role in controlling immune surveillance even in a quiescent state (22–24). Further, during inflammation ECs become activated, modulating blood flow and vascular permeability, creating new vasculature through angiogenesis, and promoting leukocyte TEM, primarily at post-capillary venules. To assist in TEM, ECs upregulate expression of leukocyte adhesion molecules (LAMs), such as selectins and cell adhesion molecules (CAMs) that interact with complimentary molecules on circulating immune cells to slow them down and guide entry into the tissue [reviewed in (25, 26)]. Generally, selectins enable rolling of immune cells across the endothelium to slow them down, and CAMs are used for firm arrest and then extravasation of the immune cell across the EC layer, with aid for extravasation from adhesion molecules such as platelet endothelial cell adhesion molecule-1 (PECAM-1). However, the expression of these molecules by ECs varies between tissues, and the mechanisms of migration are tissue-dependent (Figure 1) (18, 19, 27, 28). While EC heterogeneity is well-acknowledged, only recently have tissue-specific characteristics and functions been studied in detail, and only in a few organ systems, such as the brain, lung, kidney, and liver. Since immune cell interactions with endothelium depend on disease states and localization, and adhesive molecules serve as potential therapeutic targets, incorporating tissue-specific ECs in *in vitro* models of inflammation is important for the models to predict effective treatment strategies (29).

**Figure 2:**
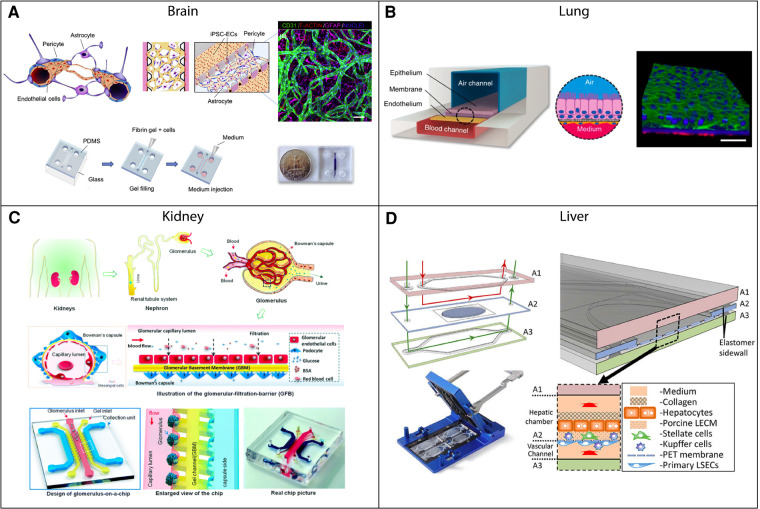
Examples of vascularized tissue chips for different organ systems. (**A**) Tissue chip example of the blood-brain barrier (BBB). The 3D-BBB contains a microvascular network that forms through a fibrin gel *via* vasculogenesis. Adapted from (31) with permission; copyright Elsevier Science & Technology Journals. (**B**) Small airway-on-a-chip example. The tissue chip contains blood and air channels separated by a porous membrane. Pulmonary microvascular endothelial cells line the blood channel and are exposed to fluid flow. Adapted from (32) with permission; Copyright © 2015, Nature Publishing Group, a division of Macmillan Publishers Limited (CC-BY). (**C**) Example of a glomerulus-on-a-chip. The chip contains capillary, gel, and collection channels, with glomerular endothelial cells cultured in the capillary channel and exposed to fluid flow. Adapted from (33) with permission from Royal Society of Chemistry. (**D**) Tissue chip example of the liver. Liver sinusoidal endothelial cells and Kupffer cells are cultured in a vascular chamber, which is separated from a hepatic chamber by a porous membrane. Both chambers have fluid flow rates that are representative of the flow experienced *in vivo*. Adapted from (34) with permission from the Royal Society of Chemistry.

As organ-specific functions of the vasculature develop from both stable epigenetic programming and environmental signals, careful thought must be given both to cell sources and the microenvironment these cells are cultured in when modeling a given tissue system. The development and application of tissue chip technology (a.k.a. “microphysiological systems” or “organ-on-chips”) provides researchers the opportunity to more faithfully recapitulate specific microenvironments for *in vitro* models. They do so by recreating both the structure and function of an organ or organ unit, often incorporating multiple key cell types and mechanical stimuli. They enable a reductionist approach to probe at disease mechanisms or make more accurate predictions of drug efficacy or toxicity prior to human clinical trials. By using patient-derived cells, tissue chips have the potential for personalized medicine. Several foundational studies have illustrated the ability of these models to accurately predict drug toxicity and drug and vaccine efficacy (30). More recently acknowledged has been the importance of vascularizing these tissue chip models. Several methods of vascularization have been developed, and we refer readers to these reviews for details on these techniques (3, 6). Further, different vascularization strategies and tissue chip configurations have been used to model a variety of critical organs (Figure 2). Many models thus far have relied heavily on the readily available and easy-to-culture human umbilical vein EC (HUVEC) line. However, given the importance of the endothelium in disease and the heterogeneity of endothelial cells and their inflammatory responses, tissue chip models should start to incorporate tissue specific ECs, as well as critical support cells, when possible.

## Endothelial cells

### Background

Endothelial cells are a heterogenous population that demonstrate high levels of organ-specific structure and function (summarized in Figure 1, Table 1), emerging from both epigenetic specification and environmental influences. EC heterogeneity was first recognized in the 1980s as a fundamental characteristic of the endothelium, appearing in the vasculature of the oldest extant vertebrate, the hagfish (35, 36). Differences between ECs include cell shape, junctional protein expression, endocytic and transcytosis levels and pathways, and glycocalyx composition. Further, ECs can be classified broadly into three categories: (1) fenestrated, (2) discontinuous, and (3) continuous (19, 37). Fenestrated cells are found in organs where filtration or secretion are primary functions of the vasculature, such as the kidney and liver. Similar to fenestrated endothelium, discontinuous endothelial cells have fenestrations that are larger in diameter and lack a diaphragm, appearing as gaps in the cell. Discontinuous ECs also have poorly developed basement membranes. The sinusoidal vascular beds of the liver are one example that contains discontinuous cells. In contrast, in the brain, brain microvascular ECs (BMECs) serve a critical role in protecting the tissue from fluctuations in blood composition and, as such, are continuous. Other continuous endothelial cells are found in the skin, lung, heart and muscle. Continuous ECs are often adhered to each other *via* tight junctions, in addition to adherens junctions, to limit passage of small molecules.

**Table 1:** Endothelial Cell characteristics at sites of leukocyte transmigration.

Organ: Barrier	EC Type/BM Characteristics	EC : PC ratio	Primary TEM location	Basal LAM expression	Inflammatory LAM expression
Brain: Blood-Brain Barrier	Continuous/BM present	1 : 1 to 3 : 1	Post-capillary venules	• Very low overall • PECAM-1 • ALCAM-1 • ICAM-2 • MHC Class I	• Upregulates P-selectin (not stored in Weibel-Palade bodies), E-selectin • Upregulates ICAM-1, ICAM-2, VCAM-1, ALCAM-1
Lungs: Air-Blood Barrier	Continuous/thin BM	7 : 1 to 9 : 1	Capillaries	• PECAM-1 • ICAM-1, ICAM-2	• Upregulates P-selectin and E-selectin, but not required for rolling • Upregulates ICAM-1, MHC Class II
Lungs: Bronchial Capillary Barrier	Continuous/thin BM	7 : 1 to 9 : 1	Post-capillary venules	• PECAM-1 • E-selectin, P-selectin • ICAM-1, ICAM-2, VCAM-1	• Upregulates P-selectin and E-selectin • Upregulates ICAM-1, VCAM-1
Kidney: Glomerular Capillaries	Fenestrated/BM present	2.5 : 1	Capillaries	• PECAM-1 • ICAM-1, ICAM-2 • Low MHC Class I and II	• Upregulates E-selectin, relies on platelets for P-selectin, rolling under debate • Upregulates ICAM-1, VCAM-1, MHC Class I and II
Liver: Liver Sinusoids	Discontinuous/no organized BM	10 : 1	Capillaries, some at post-capillary venules	• JAM-A • ICAM-1, ICAM-2, VAP-1 • MHC Class I and II	• Does not require selectins but subset can upregulate E-selectin • Upregulates ICAM-1, VCAM-1, VAP-1, MHC Class II

EC, endothelial cell; BM, basement membrane; PC, pericyte; TEM, transmigration; LAM, leukocyte adhesion molecule; PECAM-1, platelet endothelial cell adhesion molecule-1; ALCAM-1, activated leukocyte cell adhesion molecule-1; ICAM-1, intercellular adhesion molecule-1; ICAM-2, intercellular adhesion molecule-2; VCAM-1, vascular cell adhesion molecule-1; MHC, major histocompatibility complex; JAM-A, junctional adhesion molecule-A; VAP-1, vascular adhesion protein-1.

While the mechanisms driving vascular heterogeneity are still not fully understood, it is clear that some tissue specificity emerges from cell-intrinsic developmental pathways that are epigenetically regulated (12–14, 36). Certain genes persist for several passages of *in vitro* cell culture and are thus, mitotically stable (13, 18, 38). For example, in a study of lung EC cultures, the majority of studied protein persisted in culture, while several proteins expressed *in vivo* were lost (17). Microarray profiling and bulk and single cell RNA sequencing (scRNAseq) have identified transcription factors that regulate these tissue specific expression patterns. GATA Binding Protein 4 (GATA4) upregulates sinusoidal endothelial genes in hepatic endothelial cells (14, 39, 40), and mesenchyme homeobox 2 (MEOX2) and transcription factor 15 (TCF15) are critical regulators in heart ECs to mediate fatty acid uptake (41). Interestingly, new evidence from RiboTag transgenic mouse studies illustrate that tissue-specific ECs also express markers thought to be specific to other cell types within that tissue (27). For example, the authors demonstrated that brain ECs express canonical neuronal markers, and heart endothelium expresses markers thought to be restricted to cardiomyocytes. To exclude non-EC contamination, the authors crosschecked their data with two scRNAseq datasets and confirmed these findings.

Environmental cues clearly contribute to the unique phenotypes of endothelial cells along the regions of vasculature and in different tissues (15, 16). This is most clearly demonstrated in *in vitro* studies, where removal of ECs from their *in vivo* environment results in dedifferentiation or phenotypic drift of the primary cells. For example, when BMECs are isolated from the brain and cultured *in vitro*, they form more permeable barriers than seen *in vivo*. Addition of support cells, such as pericytes and/or astrocytes, or conditioned media from these cells, to the microenvironment can help recover some of the lost functionality (42). Other stimuli include contact-dependent cell communication and mechanical stimuli such as shear stress, cyclic strain, matrix stiffness, and curvature (6, 43). The mechanical stresses an EC encounters will differ depending on tissue location (12, 43). Further, ECs in certain tissues can be exposed to extreme environments, such as high oxygen across the blood-air barrier in the lungs and hypoxic conditions in the kidney (12, 36). Therefore, environmental factors driving particular EC phenotypes include paracrine signals, interactions with matrix, and mechanical factors. Any *in vitro* environment that fails to reconstitute these factors is likely to experience shortcomings as a tissue-specific model, however, tissue chip platforms help mitigate some of these shortcomings. For example, the dynamic *in vitro* blood–brain barrier (DIV-BBB) is designed in a tube structure to add the curvature to the ECs and fluid flow (44). Other vascularized chip models include post-capillary venule expansions (45), many incorporate physiologically-relevant hydrogels [reviewed in (46)], and most have fluid flow capabilities to mimic the shear stresses from blood flow experienced by endothelial cells (31, 34, 44, 47–54). Addition of these environmental cues improves endothelial cell permeability (55), viability (56), and cytoskeletal architecture (57). Incorporation of extracellular matrix (ECM) in tissue chip models helps enable cell attachment, guide cell function, and establish EC polarization (46). Further, tissue chips incorporating hydrogels or other ECM matrices (e.g., fibrin matrices) can be used for development of perfusable, physiological vascular networks *via* vasculogenesis and/or angiogenesis (31, 47, 48). Finally, the consequences of modulating the microenvironment can be thoroughly evaluated on tissue chip platforms. For example, disturbed flow experienced by ECs in atherosclerotic lesions has been investigated on a tissue chip platform. Wang and colleagues discovered the critical role of Yes-associated protein (YAP)/transcriptional coactivator with PDZ-binding motif (TAZ) activation in promoting the proinflammatory EC phenotype seen in these lesions (58). Therefore, tissue chips enable researchers to evaluate the role of environmental cues on EC function in conditions of health and disease.

### “Generic” endothelial cells

The first isolated human endothelial cells came from umbilical veins (HUVECs). HUVECs are easy to access and culture, express many key EC markers, junctional proteins and inflammatory proteins, and have served as a robust line for many scientific discoveries (6). Because of these conveniences, HUVECs are often used as a “generic” EC in models of various tissues (Table 2). As one example, HUVECs were incorporated into a liver biochip to identify a potential new biomarker for sepsis, CAAP48, which is found in higher concentrations in sepsis patients and appears to contribute to liver dysfunction during sepsis (59). In this study, HUVECs were cultured with differentiated HepaRG hepatocytes in MOTiF biochips. While immune cell migration was not directly evaluated, Blaurock-Möller and colleagues did measure release of soluble intercellular adhesion molecule-1 (sICAM-1) and vascular cell adhesion molecule-1 (sVCAM-1) after treatment with CAAP48. Both are shed during sepsis and contribute to leukocyte migration. The study found that CAAP48 led to increases in both sICAM-1 and sVCAM-1 and may contribute to the uncontrolled inflammatory response during sepsis. Our lab has also utilized HUVECs to create a **m**icro**v**ascular **m**imetic tissue chip model, termed (µSiM-MVM). HUVECs were cultured on ultrathin, nanoporous, optically-transparent membranes to monitor neutrophil transmigration. One study found that neutrophil TEM may cause small, local increases in EC layer permeability (60), while the other probed at directional stimulation and its effects on TEM (61). In the latter study, ICAM-1 reorganization was visualized on the EC surface following tissue-side, or abluminal, stimulation, likely in order to capture neutrophils and guide extravasation to the underlying tissue.

**Table 2:** Endothelial cell sources for tissue models.

Organ	Cell Source	EC Classification	Main Challenges	Evaluated LAM Expression	Tissue Chip Models with TEM
“Generic” EC	HUVEC	Continuous	Macrovascul-ature, not tissue-specific	• Expresses/upregulates key molecules	• (59) • (60) • (61)
	hMVEC	Continuous	Not tissue-specific	• Expresses/upregulates key molecules	• (62)
	BOEC	Continuous	Not tissue-specific, variable success rate for isolation and culture	• Expresses/upregulates key molecules	• None
	hiPSC: reviewed in Jang et al., 2019; Williams and Wu, 2019; Xu et al., 2019	Continuous	Not tissue-specific, immature phenotype	• Variable • Upregulates ICAM-1 • Fails to upregulate VCAM-1 (Orlova et al., 2014)	• (50) (adhesion only)
	hiPSC: cord blood-derived CD34^+^	Continuous	Not tissue specific	• Upregulates ICAM-1, ICAM-2, VCAM-1	• (63) (for brain)
	hiPSC (rEC): Lu et al., 2021b	Continuous	Not tissue specific	• Expresses PECAM-1 • Upregulates E-selectin	• None (proposed for future brain models)
	hiPSC: viEC	Continuous	Not tissue specific	• Did not evaluate	• (64) (for glomerulus)
BMEC	Immortalized Human (hCMEC/D3)	Continuous	Rapid de-differentiati-on	• Expresses/upregulates key molecules	• None
	Primary Human	Continuous	Difficult to isolate, rapid de-differentiation	• Expresses/upregulates key molecules	• (44) • (65)
	hiPSC (iBMEC): Lippmann et al., 2012	Continuous	Endothelial/epithelial hybrids	• No expression of E-selectin, P-selectin, ICAM-2, VCAM-1	• None
	hiPSC (EECM-BMEC): Nishihara et al., 2020	Continuous	Weaker barrier properties	• Expresses PECAM-1 • Upregulates P- selectin, E-selectin • Upregulates ICAM-1, ICAM-2, VCAM-1	• (66)
Lung EC	Primary Human (HMVEC-L, LMVEC, HLVEC, and HPMEC)	Continuous	Mix of blood and lymphatic ECs, genetically heterogenous, slow-growing, limited passages	• Upregulates E-selectin • Upregulates ICAM-1, VCAM-1	• (51) • (32) • (67)
	hiPSC: Taniguchi et al., 2020	Continuous	Immature phenotype	• Did not evaluate	• None
GEC	Primary Human	Fenestrated	Difficult to isolate and culture, limited lifespan	• Upregulates E-selectin (Cell Systems)	• None
LSEC	Immortalized Human (TMNK-1)	Continuous	Rapid dedifferentia-tion, chronically activated	• Upregulates key molecules	• None
	Primary Human (HLEC)	Fenestrated	Mix of human liver derived ECs (80% LSECs)	• Upregulates ICAM-1	• (34)
	hiPSC: Koui et al., 2017; Gage et al., 2020	Fenestrated	Not fully characterized	• Did not evaluate	• None

EC, endothelial cell; LAM, leukocyte adhesion molecule; TEM, transmigration; BMEC, brain microvascular endothelial cell; GEC, glomerular endothelial cell; LSEC, liver sinusoidal endothelial cell; HUVEC, human umbilical vein endothelial cell; hMVEC, human microvascular endothelial cell; BOEC, blood outgrowth endothelial cell; hiPSC, human induced pluripotent stem cell; rEC, *EEF*-reprogrammed Epi-iBMECs; viEC, vascular endothelial cell; hCMEC/D3, human cerebral microvascular endothelial cell; iBMEC, induced brain microvascular endothelial cell; EECM-BMEC, extended endothelial cell culture method brain microvascular endothelial cell; HMVEC-L, human microvascular endothelial cell-lung; LMVEC, lung microvascular endothelial cell; HLVEC, human lung vascular endothelial cell; HPMEC, human pulmonary microvascular endothelial cell; TMNK-1, immortalized human-liver endothelial cell line with SV40T and hTERT; HLEC, human liver-derived endothelial cell; PECAM-1, platelet endothelial cell adhesion molecule-1; ICAM-1, intercellular adhesion molecule-1; ICAM-2, intercellular adhesion molecule-2; VCAM-1, vascular cell adhesion molecule-1.

**Figure 3:**
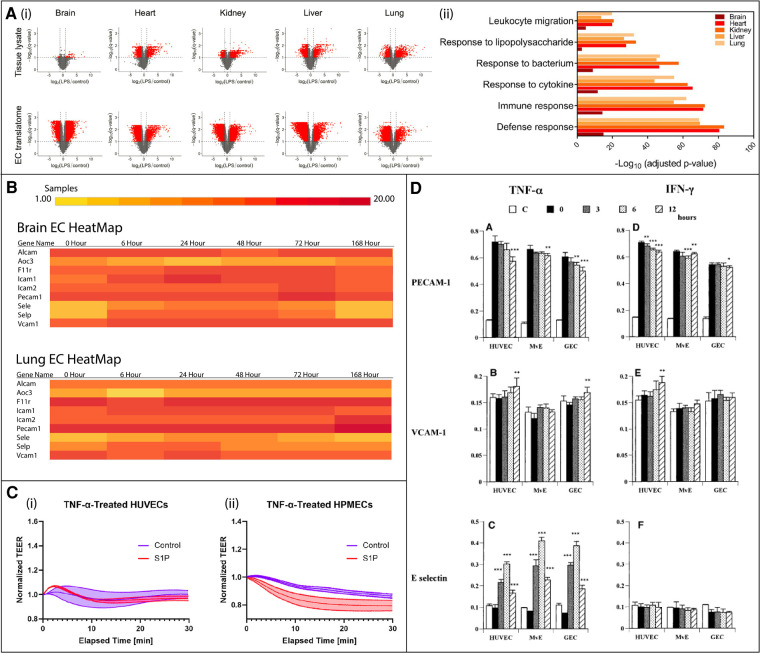
*In vivo* murine and *in vitro* human studies illustrating endothelial cell (EC) heterogeneity in response to inflammatory stimuli. (**A**) *In vivo* murine EC-translating ribosome affinity purification (TRAP) study highlights tissue heterogeneity as demonstrated by variable upregulated and downregulated transcriptomes and translatomes following LPS exposure *via* volcano plots (i) and GO analysis (ii). Leukocyte migration responses vary by tissue. Adapted from (15), used under Creative Commons PNAS license. (**B**) *In vivo* RiboTag transgenic mouse study shows differentially expressed genes in brain and lung ECs in response to LPS. The time course response varies between tissues. Created at http://www.rehmanlab.org/ribo from open access data generated by (27), under Creative Commons Attribution License (CC BY 4.0). (**C**) *In vitro* comparison of the response of two human cell lines, human umbilical vein ECs (HUVECs) and human pulmonary microvascular ECs (HPMECs), to TNFα stimulation with or without EC permeability regulator sphingosine-1-phosphate (S1P). While HUVECs are desensitized to TNFα following S1P exposure (i), HPMEC barriers are further disrupted (ii). Adapted from (68), used under Creative Commons CC BY-NC-ND license. (**D**) *In vitro* comparison of the response of three human cell lines, HUVECs, glomerular endothelial cells (GEC), and dermal microvascular endothelial cells (MvE), to TNFα and IFNγ stimulation. The kinetics of the expression of leukocyte adhesion molecules, PECAM-1, VCAM-1, and E-selectin, significantly differs between cell sources. Adapted from (28) with permission from Elsevier; Copyright © 2001 Academic Press.

Despite the conveniences of HUVECs, an obvious limitation of this cell line is that they are derived from the macrovasculature, which have mechanical, structural, and functional differences compared to the microvasculature (38). In a comparison of LAM expression kinetics between HUVECs, glomerular endothelial cells, and dermal microvascular endothelial cells, each cell type had significant differences in response to tumor necrosis factor-α (TNFα) and interferon gamma (IFNγ) stimulation (Figure 3) (28). Another recent study from our lab demonstrated differential barrier responses of HUVECs compared to human pulmonary microvascular endothelial cells in response to barrier modulating molecule, sphingosine-1-phosphate (Figure 3) (68). Both studies highlight the limitations of this model for representing tissue-specific vascular barriers. Further, immune cell trafficking and most tissue-specific heterogeneity occurs at the level of the microvasculature (22, 25, 26). A common alternative to HUVECs are commercially-available human microvascular endothelial cells (hMVEC) (Table 2). hMVECs can come from several sources but are commonly derived from the dermis. This cell line was used in a tissue chip model designed to study mechanisms of neutrophil transmigration (62). By creating chemotactic gradients, the group was able to model *in vivo*-like neutrophil TEM across the hMVEC layer and through a collagen gel to the “wound” chamber. They discovered that in the absence of an endothelial layer, neutrophils did not migrate as far, indicating EC-neutrophil interactions assist in neutrophil migration to the wound site. While there are likely benefits to using hMVEC over HUVEC to model the microvasculature, neither cell type fully recapitulates the tissue-specificity desired for tissue chip models.

Another alternative “generic” EC source is blood outgrowth ECs (BOECs) (Table 2). BOECs are endothelial progenitors isolated from the blood using density gradient isolation and can be matured in culture to create patient-specific EC models (69–72). Their gene expression is similar to HUVECs, and they are responsive to shear stress and cytokine stimulation (73). Recent studies have incorporated BOECs into tissue chip platforms. For example, Mathur and colleagues developed an arteriole “vessel-chip” using BOECs, which upregulated ICAM-1 after TNFα stimulation (74). The vessel-chips were also supportive of platelet adhesion and increased small molecule permeability upon TNFα treatment. In another study led by Mathur, BOECs were isolated from patients with sickle cell disease (SCD) and cultured in microfluidic vessel-chips (49). Both SCD patients exhibited upregulation of E- and P-selectin, as well as more moderate upregulation of ICAM-1 and VCAM-1. While neither of these studies tested immune cell migration, given BOEC's expression of cell adhesion molecules, these studies are feasible. While BOECs are a promising, patient-specific EC source, there has been variability in the success rate of BOEC cultures, with some individuals appearing unable to support BOEC isolation and culture (70, 75). Future studies would be needed to optimize this technique.

Another patient-specific “generic” EC source is human induced pluripotent stem cell (hiPSC)-derived ECs. Several protocols have been developed that produce CD31^+^, CD34^+^, and/or von Willebrand factor (vWF)^+^ endothelial cells, which have been described in prior reviews (Table 2) (76–78). Most protocols transition the cells initially through a mesodermal lineage using an embryoid body approach, 2D approach, co-culture or 3D culture techniques. Following these steps, a variety of methods have been developed to drive cells towards an endothelial phenotype. While many EC differentiation strategies are limited by low yield and require a purification step *via* magnetic-activated cell sorting (MACS), more efficient methods have recently been developed using bone morphogenetic protein 4 (BMP4), transforming growth factor-β (TGF-β), or transcription factor approaches (76, 77, 79). Several of the methods for generating hiPSC-ECs have been evaluated for their inflammatory response phenotypes. For example, Adams and colleagues’ embryoid body technique produces cells which express E-selectin, ICAM-1, and VCAM-1 in a time-dependent manner when stimulated with proinflammatory cytokines, TNFα and interleukin-1β (IL-1β), and bacterial antigen, lipopolysaccharide (LPS) (80). Further, neutrophil and T cell transmigration was comparable to HUVECs. Another embryoid body approach to generate hiPSC-ECs cultured these cells in a microfluidic device and found increased monocyte adhesion with TNFα stimulation (50).

Despite recent advances, hiPSC-derived ECs appear to be developmentally immature compared to primary ECs (81). In a side-by-side comparison of CD31^+^ and CD34^+^ hiPSC-ECs generated using Orlova et al.'s method (82), HUVECs, and human dermal blood ECs (HDMECs), the inflammatory responses of each cell differed significantly (81). While all cells upregulated some adhesion molecules, such as ICAM-1, in response to TNFα, hiPSC-derived ECs lacked upregulation of VCAM-1. hiPSC-derived ECs also promoted less immune cell adhesion compared to HUVECs. Interestingly, CD31^+^ hiPSC-derived ECs formed tighter barriers and had a more robust inflammatory responses compared to CD34^+^ hiPSC-derived ECs. It would be beneficial to have similar comparisons for other stem-cell derived ECs, as understanding their inflammatory phenotypes could help researchers decide which method to employ for particular models and/or hypotheses. In addition, while baseline characterization of inflammatory responses is critical, technical challenges have hindered validation of the inflammatory response in the context of specific tissue microenvironments. It is not unreasonable to think that developmentally immature ECs may progress towards a more mature phenotype when cultured within a tissue chip environment containing critical support cells and environmental signals. In fact, a recent BMEC-like cell differentiation protocol which differentiates hiPSCs into endothelial progenitor cells and sorts for CD31^+^ cells before specifying a brain microvascular EC-like phenotype, also did not see upregulation of VCAM-1 on BMEC-like cells grown as a monoculture (83), similar to the results of CD31^+^ ECs generated *via* the Orlova method. However, VCAM-1 upregulation was rescued by coculture with smooth muscle-like cells (SMLCs) or SMLC-conditioned medium. Future studies, therefore, could characterize “generic” ECs cultured within tissue chips for further differentiation and mature inflammatory phenotypes.

### Brain

The capillary network within the brain is the tightest in the body. The blood-brain barrier contains continuous endothelial cells zipped together by tight junction molecules, in contact with pericytes embedded within a basement membrane and nearby astrocyte endfeet. These cells have very low rates of transcytosis and upregulate expression of several transporters, such as glucose transporter GLUT1 and ABC transporter MDR1, to supply the underlying brain tissue with necessary nutrients (84). RiboTag^EC^ murine data indicates that a large portion of enriched genes in BMECs are involved in transport, including ion, acid, and neurotransmitter transport (27). Breakdown of this barrier is a common feature of several cognitive dysfunctions, including Alzheimer's disease (AD), Parkinson's Disease, and Multiple Sclerosis (MS) (85–88). Traditionally thought of as “immune privileged” the blood-brain barrier does allow immune cell migration during inflammation, as well as low level entry of T cells in healthy conditions for surveillance purposes (89–91). This process is aided by constitutive expression major histocompatibility complex (MHC) class I molecules by BMECs. However, expression of other adhesion molecules during homeostasis is lower than peripheral ECs (92). The brain endothelium responds to inflammatory stimuli with upregulation of LAMs, allowing immune cells to cross the BBB using the traditional mechanisms of rolling, arrest, and TEM (27, 89, 93). P-selectin, however, is not stored in Weibel-Palade bodies on brain endothelial cells, leading to delayed surface expression and slowed recruitment of leukocytes during inflammation (94). Interestingly, different regions of the brain express varying levels of adhesion molecules, and thus, immune cell migration differs between brain regions (90). Further separating BMEC inflammatory responses from other tissue-specific ECs, brain endothelium appears to upregulate, rather than downregulate, more of its ribosome associated transcripts in response to LPS (Figure 3) (15). The consequences of this increase in actively translated mRNAs, however, have not been studied further.

Given that the BBB is implicated in numerous diseases and is a critical target for drug delivery, many tissue chip models of this barrier have been developed (65, 95–99). Because the unique properties of BMECs are long recognized and well-studied, these models rarely settle for generic endothelial cells and the topic of BMEC source has been reviewed in depth (100–102). The most common cells lines incorporated into *in vitro* tissue chip models have been human primary cells and stem cell-derived brain ECs (Table 2). Human primary brain ECs are difficult to obtain, and immortalized lines, such as hCMEC/D3, are notorious for dedifferentiation in culture and weaker barrier properties compared to *in vivo* barriers. However, incorporation of primary cells into tissue chips systems, in particularly under flow conditions and in coculture with pericytes and/or astrocytes, has been shown to improve these phenotypes (42, 103). Commercially available primary human brain ECs were incorporated into the DIV-BBB within hollow fibers containing a mixture of 0.2 to 0.5 µm pores and ≈2 to 4 µm pores to allow for immune cell migration across chambers (44). As the fibers were designed to mimic the diameter of the microvasculature, the platform serves as a potential model to directly study immune cell transmigration mechanisms. The group demonstrated that monocytes crossed the barrier during flow cessation followed by reperfusion, and the barrier experienced a biphasic opening, as seen in prior *in vivo* studies. In another model, primary human BMECs were cultured in a blood-brain barrier-on-a-chip (B^3^C) in vascular channels, with 3 µm pores for leukocyte migration (65). The set up enabled real-time analysis of neutrophil migration, discovering that protein kinase C-delta inhibits interaction of neutrophils with endothelial cells, reducing both adhesion and migration. Therefore, primary human brain ECs provide a facile alternative for those interested in studying immune cell migration mechanisms despite their weak barrier properties compared to their *in vivo* counterparts.

One of the first human alternatives to primary cells were umbilical cord blood (UBC)-derived CD34^+^ ECs, which obtain BBB-like properties when cocultured with pericytes (104, 105). Mossu and colleagues utilized these cells in a microfluidic cerebrovascular barrier model (µSiM-CVB) to study T-cell TEM under flow (63). Upon confirmation that the cells expressed ICAM-1, ICAM-2, and VCAM-1 upon TNFα stimulation, the tissue chip platform was used to image all steps of the TEM cascade using live cell imaging. However, this cell source is not sufficiently BBB-specific, and thus the field has turned its efforts to developing hiPSC-based BMECs.

Differentiation protocols for BMECs have undergone several iterations, including an early protocol that produced hybrid endothelial/epithelial cell phenotypes (102, 106–108). Despite these challenges, stem cells continue to be the cell of choice for many BBB tissue chips (Table 2). BMEC differentiation generally starts similar to other EC differentiation protocols, transitioning through a mesodermal state. The first hiPSC-based protocol developed co-differentiated endothelial cells and neurons, following the embryonic developmental process (109). Future iterations transitioned from undefined to defined medium and introduced retinoic acid to improve barrier properties (110). While the cells were well characterized for their brain-like phenotypes in terms of transporters and tight barrier properties, it was soon discovered that they lacked several key EC characteristics, including expression of selectins, ICAM-2 and VCAM-1, as well as expressing several epithelial cell markers (83, 102). Recently, a few responses have emerged. Lu and colleagues produced phenotypic endothelial cells (rECs) by overexpression of EC-specific transcription factors, however, the cells have not yet been cultured to produce a brain-specific phenotype. They are confirmed to increase E-selectin expression upon TNFα stimulation (111). Another novel protocol was produced by Nishihara and colleagues, which uses an endothelial progenitor cell differentiation method before specifying a brain-like phenotype through an extended endothelial cell culture method (EECM) with brain-based supplement B27 and human fibroblast growth factor 2 (83). Cells produced with this method (known as EECM-BMEC-like cells) express key inflammatory molecules. Excitingly, EECM-BMEC-like cells derived from MS patients demonstrated increased expression of both ICAM-1 and VCAM-1 at baseline and upon proinflammatory stimulation compared to healthy controls. The model was further confirmed to mimic other key MS phenotypes, including disrupted tight junctions and increased interactions with immune cells, and opens the door for testing new drugs and therapeutic approaches (112). Further, our group recently incorporated EECM-BMEC-like cells into a tissue chip platform known as the modular-µSiM (m-µSiM) (66). m-µSiM culture was confirmed to mimic the baseline and inflammatory phenotypes of EECM-BMEC-like cells cultured in traditional culture plates and Transwell systems, and supported studies of both neutrophil and T cell TEM. While the new protocols do not achieve the barrier tightness of some previous BMEC differentiation methods, they represent valuable alternatives for groups interested in studying disease mechanisms involving immune cell migration.

### Lung

Microvascular endothelial cells of the lung can be broadly classified as pulmonary or bronchial and are another example of continuous ECs. The site of gas exchange occurs at the alveolar-capillary interface, or air-blood barrier, within the pulmonary circulation, where ECs and epithelial cells are separated only by a thin basement membrane (18). Alveolar capillary ECs have several defining factors, separating them from other ECs. For example, the entire population of alveolar capillary ECs express angiotensin I–converting enzyme, in comparison to only 10% of systemic ECs (18). Recent studies have subclassified alveolar capillary ECs into “aerocytes” (aCap) and “general” capillary (gCap) (113). The main function of aerocytes is gas exchange and leukocyte migration, whereas gCaps serve roles in vasomotor tone, capillary homeostasis, and repair. Interestingly, aerocytes lack expression of major constituents of Weibel-Palade bodies specific to ECs, such as vWF, P-selectin, and endothelin 1 (EDN-1). The thinnest of lung capillaries, where gas exchange takes place, are where Weibel-Palade bodies are known to be absent (114, 115). They do, however, express other common EC markers, such as PECAM-1 and CD34 (13, 18).

There are unique aspects of the air-blood barrier that make it a site of high leukocyte transmigration. Transmigration does not appear to involve selectin mediated rolling and occurs in the small pulmonary capillaries, instead of post-capillary venules (18, 116, 117). Neutrophil deformation leads to sequestering in the capillaries, and then LAMs such as ICAM-1 keep the neutrophils in place. In addition, P-selectin can contribute to leukocyte sequestration, and upregulation of P-selectin and E-selectin are critical markers of lung inflammation (29, 118). While lung ECs significantly upregulate genes related to leukocyte adhesion and migration (Figure 3), T cell activation, and regulation of immune system processes in response to LPS, at least in mice, this response is delayed compared to the responses of brain and heart ECs (27). Additionally, *Car4*-high ECs have recently been identified in mice using single cell RNA sequencing (119). *Car4*-high ECs express high levels of Car4, CD34, and VEGF receptors, and localization and proliferation is enriched at sites of influenza-induced lung injury in the alveolus. *Car4*-high ECs are primed to receive VEGFA signals from damaged alveolar type I epithelial cells for repair and regeneration, as it has been suggested that *Car4*-high ECs may play a role in vasculogenesis. Bronchial capillary ECs, on the other hand, are less well studied and therefore poorly understood. Although less leukocyte TEM occurs in the bronchial compared to pulmonary capillaries, bronchial ECs constitutively express E-selectin and P-selectin (18, 120). This is likely due to constant exposure to antigens. They can upregulates selectins, ICAM-1 and VCAM-1 under inflammation (117). Future studies characterizing these cells will be critical, as they are implicated in several diseases, including chronic obstructive pulmonary disease (COPD) and SARS-CoV-2 (32, 121).

Most of the discussion around cell sources for lung tissue chips has focused on lung epithelial cells, however a few recent reviews have discussed EC sources in *in vitro* lung models (113, 122, 123). Endothelial cells used in tissue chip models thus far have largely been HUVECs and primary human lung microvascular ECs (HMVEC-L, LMVEC, HLVEC, or HPMEC; Table 2) (32, 51, 67, 123–125). Primary lung ECs are available commercially and often obtained during biopsies. As with most primary human cells, they risk having a diseased phenotype, may be pooled and therefore genetically heterogenous, are slow-growing, and have a limited passages (122–125). In addition, commercial sources are now specifying that the available lung microvascular ECs are a mix of lymphatic and vascular ECs, as it is extremely challenging to distinguish between the two cell types (32). Despite these limitations, they upregulate E-selectin, ICAM-1, and VCAM-1 in response to various proinflammatory stimuli (123). Primary lung microvascular ECs have been used in lung tissue chip models of both alveolar-capillary interface and bronchial capillaries (32, 51, 67). The seminal tissue chip study was a lung-on-chip model, incorporating alveolar epithelial cells with HLVECs. They observed upregulation of ICAM-1 in response to TNFα stimulation, along with secretion of cytokines by HLVECs (51). This tissue chip was later modified to model a human “small airway-on-a-chip” to study asthma and COPD (32). The model contained commercially-available healthy control and COPD donor primary human airway epithelial cells in one chamber and commercial human lung microvascular endothelial cells in the opposite chamber. The epithelial cells were first differentiated into a bronchiolar epithelium, composed of the many cell types found within that layer. The model was confirmed to mimic drug responses *in vivo* in terms of neutrophil recruitment and LAM expression after stimulation with viral mimic poly(I:C) or LPS. When testing a drug with known limited activity in COPD patients, there was no change in neutrophil adhesion or expression of E-selectin, ICAM-1, or VCAM-1 upon drug administration. However, an experimental drug reduced expression of all three molecules, corresponding to reduced neutrophil adhesion to the endothelial layer. Importantly, this was only observed in the flow conditions of the lung tissue chip, and not in a Transwell™ model, highlighting the importance of mechanical stimuli to EC function. Another bronchiole lung tissue chip by Barkal and colleagues consisting of primary human bronchial epithelial cells, pulmonary fibroblasts, and LMVECs was used to characterize the bronchiole inflammatory response to fungal infection (67). The group analyzed polymorphonuclear leukocyte (PMN) migration across the vasculature and toward the *Aspergillus fumigatus* (*A*. *fumigatus*) hyphae. There was increased PMN transmigration in infected chips compared to controls, and even greater migration in a *ΔlaeA* mutant model. *ΔlaeA* mutants are known to be less virulent, likely due to reduced production of molecules used to evade the host immune system. This work indicates that greater PMN recruitment may aid in the clearance *ΔlaeA* mutants over wildtype *A*. *fumigatus*.

Given the success of various lung-on-chip models using primary human microvascular cells, as well as the ability to obtain these samples directly from patient populations, there appears to be little drive to produce stem-cell derived lung ECs. To the best of our knowledge, currently, the only *in vitro* models that have explored hiPSC-ECs in lungs have been for tissue regeneration purposes (Table 2) (126). Due to the immature cell phenotype, they fell short in several parameters compared to both HUVECs and HMVEC-Ls. Regardless, with the advantages of personalized medicine and the limitations of primary lung microvascular ECs, including lymphatic EC contamination, it may be beneficial for the field to start exploring these options.

### Kidney

Filtration within the kidney occurs in the glomerulus, which contains fenestrated endothelial cells, a basement membrane, and podocytes. Like most endothelium, glomerular ECs (GECs, also GEnC) arise from the mesoderm. However, their development occurs mainly through vasculogenesis rather than in combination with angiogenesis (127). GECs produce a robust glycocalyx and have fenestrations which are 60–80 nm in diameter, allowing selective filtration based on size and charge (18). Similar to lung ECs, GECs express normal levels of PECAM-1 and CD34, but lower levels of vWF (13, 18). Barrier function is, in part, controlled by paracrine signaling from podocytes, which wrap their foot processes around the ECs (11). While many tissue chip studies focus on filtration functions of GECs, leukocyte infiltration occurs during glomerulus inflammation and has been implicated as the cause of damage in several diseases, including diabetic nephropathy, lupus nephritis, and sepsis (128–131). Immune cell migration in the kidney is unique, as the glomerulus is a capillary-based structure and lacks post-capillary venules. However, glomerular capillaries, similar to pulmonary capillaries, are supportive of leukocyte TEM (132, 133). Further, unlike most endothelium, which stores P-selectin in Weibel-Palade bodies, GECs do not express significant amounts of P-selectin on their surface. Thus, the adhesion of leukocytes through P-selectin/P-selectin glycoprotein ligand-1 (PSGL-1) interactions in the glomerulus first requires the adhesion of platelets, an often neglected component of tissue chip models (134). GECs also constitutively express ICAM-1, and can further upregulate ICAM-1, along with E-selectin, VCAM-1, and MHC Class I and II proteins during inflammation (28, 29, 135). Despite expression of selectins on GECs, immune cell migration does not always appear to start with rolling in the glomerulus, though this has been debated (29, 136, 137).

Due to challenges in isolating and culturing primary human GECs, *in vitro* studies have often relied on mouse or rat cells (138). However, recent improvements have led to commercial availability of primary human GECs, as well as protocols for isolation of GECs from patient samples, both of which have been implemented in glomerulus tissue chip models (Table 2) (52, 53, 139). There are even commercial sources of primary human glomerular microvascular endothelial cells which have been functionally assayed for inducible expression of E-selectin (140). As with most primary cells, however, commercial and self-isolated GECs have limited lifespans. One tissue chip model was able to culture primary GECs obtained from kidneys that were “non-suitable for transplantation” - primarily from infant patients whose cause of death were not related to the kidney (141). The cells retained several key properties of GECs, including presence of fenestrations. It is likely, however, that most labs do not have access to these materials. To our knowledge, none of these models have studied immune cell trafficking into the glomerulus.

To date, there are no GEC-specific differentiation protocols, and hiPSC-derived EC incorporation into glomerulus tissue chips is limited. However, Roye and colleagues developed a personalized glomerulus tissue chip using stem cell-derived epithelium and vascular endothelium (viEC) (Table 2) (54). The group utilized a protocol by Atchison et al. that follows the same initial transition through the mesoderm, and then specifies into viEC through VEGF-A and a cyclic adenosine monophosphate (cAMP) booster (64). Cells were then sorted for CD31^+^/VE-cadherin^+^ expression. While these cells are not differentiated into a tissue-specific phenotype, co-culture with hiPSC-derived podocytes demonstrated mature functions of the glomerulus, along with a disease phenotype when treated with a nephrotoxic drug. Future studies will need to analyze the immune response of the ECs in this culture. As the first glomerulus-on-chip was developed under a decade ago, it is not surprising that studies thus far have focused on filtration properties. Hopefully as the field develops, immune cell infiltration will be incorporated into these disease models to gather a more complete picture of disease progression and identification of therapeutic targets.

### Liver

The capillaries of the liver are contained within its sinusoids. Blood arrives here from two vascular systems, the arterial and portal vasculature (18). The endothelial cells in the sinusoids are discontinuous, with their primary function being filtration. They lack an organized basement membrane and function in partner with tissue resident macrophages, called Kupffer cells, to remove wastes, ECs doing so mainly through endocytosis (11, 18). Liver sinusoidal endothelial cells (LSECs) represent a heterogenous population, with distinct phenotypes in different zones of the liver, as each zone is its own unique microenvironment (142). As the liver is a key immune organ, LSECs can present antigens to both CD4^+^ and CD8^+^ T cells *via* MHC Class I and II molecules [reviewed in (143, 144)]. Interactions between LSECs and T cells can decrease T cell activation, preventing autoimmune dysfunction throughout the body. Traditional transmigration is not always necessary for immune cell surveillance. In some instances, T cells can pass through the LSEC gaps and directly contact hepatocytes, or contact hepatocytes without crossing the LSEC barrier by contacting the hepatocyte protrusions through the fenestrations (145). Leukocytes are recruited and infiltrate the liver sinusoids in several disease condition, including hepatitis and sepsis (29). While the liver has post-capillary venules, leukocyte migration primarily occurs in the sinusoidal capillaries and does not require selectins (29, 146). However, expression of E-selectin by a small percentage of LSECs was detected in Kupffer cell (resident liver macrophages)-depleted mice and serves to recruit monocytes that replenish the Kupffer cell population (147). Another unique aspect of liver sinusoidal TEM is that junctional adhesion molecule-A (JAM-A), rather than PECAM-1, is necessary for transmigration (148, 149). LSECs also express baseline levels of ICAM-1 and vascular adhesion protein-1 (VAP-1). These, along with VCAM-1 and MHC Class II molecules, are upregulated in response to proinflammatory stimulation (146, 150, 151). Finally, LSECs can lose their fenestrations upon tissue damage (152). The complex and unique features of the liver sinusoids make *in vitro* modeling for immune cell TEM particularly challenging.

Similar to brain endothelial cells, LSECs rapidly dedifferentiate in culture, which can be partially salvaged by coculture with relevant cell types, most often hepatocytes (152, 153). Still, they are difficult to cryopreserve, and some commercially available sinusoidal ECs are derived from large vessels rather than the microvasculature (154). Commonly used alternatives have been HUVECs and human foreskin endothelium cells (HMEC-1), both of which improve hepatocyte function (155). However, neither of these cell types are discontinuous, and they lack liver-specific receptors. In fact, even immortalized LSEC line, TMNK-1, lacks fenestrations and have a chronically activated phenotype (Table 2) (155). One commercial source of LSECs is a mix of human liver derived ECs (HLECs) and reports containing up to 80% LSECs (Table 2). This source was used to develop a “Continuously Zonated and Vascularized Human Liver Acinus Microphysiological System” (vLAMPS) (34). The group designed their chip system to mimic the structure of the liver sinusoid, containing hepatocytes, Kupffer cells, and stellate cells in a hepatic chamber and LSECs in the vascular channel. The liver derived ECs formed a continuous layer with fenestrations averaging 170 nm pore diameters and demonstrated upregulation of ICAM-1 under inflammatory stimuli [1 μg/ml LPS + 15 nM epidermal growth factor (EGF) + 10 ng/ml TGF-β]. Further, fluorescently labelled PMNs could be tracked migrating across the LSEC layer and into the hepatic chamber.

Stem cell-derived LSEC protocols are still in their infancy, with the first protocol published in 2017 (Table 2) (156). Following the developmental pathway, LSEC progenitors were first generated by induction of mesodermal cells and selection of FLK1^+^CD31^+^CD34^+^ cells. Interestingly, this population already expressed several LSEC-specific genes. FLK1^+^CD31^+^CD34^+^ cells were expanded and matured by TGFβ inhibition and culture in hypoxic conditions and a LSEC signature was confirmed by looking at expression of cell-specific markers. A later publication used an embryoid body approach to induce mesodermal cells and, from there, generated arterial and venous ECs (157). Gage et al. also used TGFβ inhibition and culture in hypoxic conditions to mature the arterial and venous ECs into LSECs, but also included cAMP agonism. They discovered that the venous-like cells more readily adopted a LSEC phenotype, as evidenced by molecular, structural, and functional features, including fenestrations and scavenger capabilities. Neither the Koui et al. or Gage et al. protocols tested the inflammatory response of hiPSC-derived LSECs and to our knowledge have not been incorporated into tissue chip models. Other potential avenues for generating LSECs could utilize using newly identified transcription factors that determine LSEC fate, such as C-MAF, GATA4, and MEIS2 (39), PU.1 (encoded by the SPI1 gene) (158), and c-Maf (159).

### Musculoskeletal system

The musculoskeletal system encompasses numerous tissues with varying types and levels of vascularization. As tissue chip models of musculoskeletal tissues become more sophisticated, it is important to consider the accurate representation of vascular barriers and their roles within these tissues (160). Tissues such as muscles (161), bone (162), synovium (163), and menisci (164) are vascularized while other tissues such as tendons and ligaments (165, 166), intervertebral discs (167), and cartilage (163, 168) generally have very little or no vascularization. In these “avascular” tissues, vascularity is typically an important feature of disease states, thereby motivating the need for accurate “pathological” vascular barriers in *in vitro* models. While there are many remaining unknowns, specific EC characteristics have been identified for certain tissues.

In muscle, ECs have been shown to function in partnership with myogenic cells especially during early development. Muscle ECs secrete growth factors including insulin-like growth factor 1(IGF-1), hepatocyte growth factor (HGF), basic fibroblast growth factor (bFGF), platelet-derived growth factor-BB (PDGF-BB), and VEGF to induce myogenesis, while responding to angiogenic signals from myogenic cells (161). A tissue chip model by Osaki and colleagues recapitulated this crosstalk using light sensitive channelrhodopsin-2 (ChR2) C2C12 skeletal muscle cells (C2C12-ChR2) and HUVECs to create muscle and vascular structures in a device, which could be optically stimulated to contract (169). The group demonstrated that HUVECs responded to angiopoietin-1 secreted by muscle cells to stimulate angiogenesis. Additionally, HUVEC coculture upregulated myogenesis in muscle cells, thereby improving muscle contraction *via* angiopoetin-1/neuregulin-1 signaling.

Similarly, ECs in bone serve several important roles in development and maintaining homeostasis of surrounding tissue. Vascularization in bone includes the Haversian system of canals, which allow blood vessels to supply cortical bone, and the vasculature of the periosteum surrounding bones. Studies investigating bone ECs have identified many subpopulations that can influence osteogenesis and the formation of new blood vessels (170, 171). Additionally, ECs in the bone marrow have important traits that maintain the hematopoietic environment for production of blood cells. For example, the two main types of blood vessels found in bone marrow, arteries and sinusoids, have been shown to differentially regulate hematopoiesis. Factors including EC morphology, cell signaling, and barrier integrity influence the maintenance of stem cell or leukocyte populations (172). Bone marrow ECs have shown greater ability to induce hematopoietic progenitor cell adhesion and migration when compared to HUVECs or lung ECs. They also exhibit lower expression of vWF and constitutive expression of adhesion molecules such as VCAM-1 and E-selectin compared to other EC populations (173). Chou and colleagues created a bone marrow tissue chip using CD34^+^ and marrow-derived stromal cells to model the periarterial, perisinusoidal, mesenchymal, and osteoblastic hematopoietic niches of bone marrow (174). HUVECs were incorporated into a vascular channel. Their model was able to recapitulate pathological features, such as hematopoietic dysfunction and neutrophil maturation abnormality, when constructed using cells from patients with Shwachman-Diamond syndrome.

Tendons and ligaments together form a large category of tissue that is often considered avascular. In healthy mature tissue, vascularization is sparse and mostly limited to supportive sheath structures that provide organization, such as the endotenon or epitenon, and interfaces with muscle and bone (165, 166). While the “healthy” level of vascularization may vary among tendons and ligaments in different parts of the body, neovascularization is a common feature seen after acute injury and long-term pathological states such as tendinopathy (175). The notion that these tissues are avascular can be misleading, and it is important that *in vitro* models carefully consider the role of vasculature for their designs, particularly in disease models. Knowledge of tendon-specific ECs is still limited when compared to other musculoskeletal tissues. One tendon study has shown that resident EC populations may form a “blood-tendon barrier” that maintains a tendon stem cell niche which may have implications for tendon healing and regeneration. Further, mouse tendon vasculature was shown to form tighter barriers than cardiac vasculature, but not as tight as those found in the blood-brain barrier (176).

The endothelial cells discussed here represent some of the diversity of EC populations found throughout musculoskeletal tissues. Some ECs, such as those in bone, are better characterized than others, but all play important roles in maintaining their surrounding tissue and tissue-specific functions. Importantly, increased vascular proliferation is common in sites of injury and disease, suggesting that the notion of avascular tissues is misleading. Therefore, ECs are an important component for researchers to consider when creating *in vitro* models of musculoskeletal tissues. Most existing musculoskeletal tissue chips with vasculature components utilize HUVECs, and other sources for musculoskeletal tissue ECs are rare (160). However, accurate representation of musculoskeletal EC function will facilitate more physiologically relevant tissue systems, particularly in disease and injury models. hiPSC-derived ECs are beginning to emerge in musculoskeletal models (177), and as hiPSC techniques become more sophisticated and more tissue chip researchers move toward patient-specific models, it is likely that hiPSC-derived ECs will become increasingly incorporated into these models. Further research is needed for developing protocols to generate musculoskeletal-specific endothelial cells.

## Pericytes

### Background

Perivascular cells are support cells that line the vasculature and serve a variety of roles in both homeostasis and tissue repair (178). The three main types of perivascular cells are smooth muscle cells (SMCs), pericytes (PCs), and fibroblasts (FBs). SMCs are predominantly found along larger vessels, whereas PCs surround smaller blood vessels, including capillaries and post-capillary venules (3, 179). Cells with intermediate phenotypes are found along midsize vessels. FBs are found throughout much of the vasculature, mainly on arterioles and veins but not within capillaries (179). They are important regulators of extracellular matrix deposition and tissue remodeling after injury, along with important roles in guiding angiogenesis. As this review focuses on the microvasculature and immune cell trafficking, we will only discuss PC heterogeneity and immune functions.

Pericytes have multiple developmental lineages. While most PCs, like ECs, arise from the mesoderm, PCs of the brain and retina have a neural crest lineage (180). Mature pericytes are known to embed themselves within the basement membrane of blood vessels, where they communicate with ECs *via* gap junctions, adherens junctions, and soluble signals (180, 181). EC to PC ratio varies in different organs, with the highest coverage found in the brain and retina and lowest in skeletal muscle (Figure 1) (180, 182). Along with other mural cells, PCs aid with angiogenesis and vessel stabilization. They also regulate blood flow and have various roles in immunological defense and response to inflammation (180, 183). They are generally characterized by vessel location and morphology, which is generally stellate but varies along the vasculature and between tissues (184). Defining pericytes and other mural cells has been difficult, due to overlapping markers, but recent studies suggest inwardly rectifying potassium channel, KCNJ8, and ABC transporter, ABCC9, may be good candidates (179, 180). Traditionally, however, PCs have been identified by expression of PDGFRβ and/or NG2 (178, 180, 184).

Following TEM across the EC layer, migrating immune cells must also breach the basement membrane and pericyte layer to reach extravascular tissue. This process has been reviewed in detail prior (26, 185, 186). The resistance created by these final two layers depends on the tissue and their coverage. Pericytes have multiple roles in assisting transmigrating leukocytes during inflammation, including secretion of cytokines, remodeling and degradation of the basement membrane, and upregulation of adhesion molecules, such as ICAM-1, to guide migrating immune cells after they cross the EC barrier (187–190). Expression of LAMs, at least in some part, appears to be tissue-dependent (Figure 1, Table 3) (186). Neutrophils preferentially migrate across “hotspots,” crawling across PCs to regions with gaps in coverage or high ICAM-1 expression and regions that have low deposition of some basement membrane components (185, 189). PCs can directly interact with transmigrating immune cells before they enter the extravascular tissue. These interactions include signals that promote differentiation into immune cell subtypes (26). PCs can also damper immune responses by signaling to ECs to reduce neutrophil migration and can negatively regulate T cell responses (192–194).

**Table 3:** Pericyte sources for tissue models.

Organ	*in vivo* or primary cell LAM Expression	Cell Source	Evaluated *in vitro* LAM Expression
“Generic” PC	• N/A	hiPSC: Orlova et al., 2014; Wanjare et al., 2014; Kumar et al., 2017; Reviewed in Xu et al., 2017 and Browne et al., 2021	• Upregulates VCAM-1 (191)
Brain PC	• Baseline VCAM-1 • Upregulates ICAM-1, VCAM-1 • No selectin expression	Primary Human	• Baseline VCAM-1 • Upregulates ICAM-1, VCAM-1 • No selectin expression
		hiPSC: Faal et al., 2019; Stebbins et al., 2019	• Did not evaluate
Lung PC	• Upregulates ICAM-1, VCAM-1	Primary Human	• Upregulates ICAM-1, VCAM-1
		Immortalized Human	• Upregulates ICAM-1, VCAM-1
Kidney PC (Mesangial Cells)	• Upregulates ICAM-1 • No selectin expression	Primary Human	• Upregulates ICAM-1 • No selectin expression
Liver PC (Hepatic Stellate Cells, Ito)	• Baseline ICAM-1, VCAM-1 • Upregulates ICAM-1, VCAM-1	Primary Human	• Baseline ICAM-1, VCAM-1 • Upregulates ICAM-1, VCAM-1

N/A, not applicable; PC, pericyte; LAM, leukocyte adhesion molecule; VCAM-1, vascular cell adhesion molecule-1; ICAM-1, intercellular adhesion molecule-1; hiPSC, human induced pluripotent stem cell.

### Pericyte sources

Pericytes can be isolated from several tissues throughout the body, including the placenta, brain, heart, glomerulus, skeletal muscle and, most recently, lung (Table 3) (178, 186, 195). The characteristics of PCs from different sources vary (178). For example, brain-derived pericytes have a more elongated morphology compared to the rounded and compact pericytes found in the kidney glomerulus, also known as mesangial cells (184). Some functions appear to be tissue-specific as well. For instance, liver pericytes contribute to Vitamin A metabolism (178). In terms of immune cell migration, brain-derived PCs can upregulate both ICAM-1 and VCAM-1 upon proinflammatory stimulation and human glomerular pericytes can upregulate ICAM, but neither express selectins (26, 196–198). E-selectin was, however, expressed on human dermal pericytes derived from skin biopsies (26). Liver PCs express baseline levels of ICAM-1 and VCAM-1, both of which can be upregulated in response to TNFα (199). Both primary and immortalized human lung PCs show comparable expression of ICAM-1 and VCAM-1 in *in vitro* culture (195).

Several protocols for generating stem-cell derived pericytes have emerged in recent years. Given the variability in cell origin, some protocols transition through the mesoderm and others through the neuroectoderm. There are methods that co-differentiate ECs and PCs by generating mesodermal cells and sorting these cells to further specify and expand the EC and PC populations (Table 3) (82). Suppressing the TGF-β pathway is common in order to drive cells towards a PC-specific, rather than SMC, phenotype (82, 191, 200, 201). The characterization of the generated pericytes differs by protocol and continues to develop as the knowledge of PC and SMC phenotypes has grown. Reviews on these protocols and the phenotypic characterization of the cells generated have been previously published (3, 202). To the best of our knowledge, the only tissue-specific pericyte protocols available are two publications from 2019, both of which produce brain pericyte-like cells (Table 3). One publication by Stebbins and colleagues transitions the cells through a neural crest intermediate, while the other by Faal and colleagues presents two methods, one method transitioning cells through the mesoderm and the other *via* neural crest (200, 203). Until recently PCs have largely been ignored in *in vitro* models, likely due to the heterogeneity of this cell type and difficulty in identification of pericytes over other perivascular cells. However, several vascular barrier chips [reviewed in (4)] and BBB tissue chips (31, 204–206) have incorporated pericytes. Thus far, none of these tissue chip models have been used to study immune cell trafficking. However, one BBB model did measure adhesion molecule, VCAM-1, in hiPSC-derived pericyte-like mural cells (iMCs) differentiated from an Alzheimer's disease “APOE4/4 risk” line and an “APOE4/4-risk edited to APOE3/3-non-risk” hiPSC line (204). They discovered iMCs derived from the AD-risk cell line had increased basal VCAM-1 expression compared to the non-risk line. It is likely we will see future tissue chip studies incorporating PCs from various sources as their importance in disease continues to be established.

## Immune cells

### Background

While this review has covered the major vascular contributors to leukocyte trafficking, the sourcing of immune cells is also critical. During inflammation, an acute response is initiated by innate immune cells, first transmigrating neutrophils and then monocytes. This can turn into chronic inflammation, which involves both innate and acquired immune cells, including several types of T cells and B cells. Leukocytes migrating from the circulation into tissue can activate tissue resident immune cells such as macrophages. Discussion of the subsets of each of these immune cells is beyond the scope of this review but are detailed in many excellent books and reviews (207–209). The actions of immune cells can either lead to resolution or exacerbation of inflammation, and their inclusion into tissue chip models of disease are critical. Below, we will discuss a few subsets of immune cells and their potential cell sources.

### Neutrophils

Neutrophils, or **p**oly**m**orpho**n**uclear leukocytes (PMNs), are the most abundant white blood cell in the human body and comprise between 50%–70% of the circulating leukocyte population in healthy adults (210, 211). They are rapidly produced in the bone marrow (>10^11^ cells/day) *via* differentiation of hematopoietic stem cells (HSCs), the cell type that gives rise to all blood cells, towards granulocyte-macrophage progenitors (GMPs), ultimately resulting in mature PMNs (210, 212–214). The exact factors that promote HSC differentiation towards a terminal PMN lineage are poorly understood, and a growing body of evidence suggests that PMNs are a heterogenous population of cells with robust plasticity and polarized phenotypes, rather than a homogenous one (214, 215). This understanding emerges from multiple characterizations, which are reviewed elsewhere (210, 216–218). Circulating PMNs are thought to be short lived cells with a general half-life of 7 h postproduction from the bone marrow (219). However, this characterization is now contradicted by recent evidence suggesting that PMNs survive for longer in the body with the capability of recirculating back into vasculature and bone marrow (220, 221). Regardless, PMNs typically function by migrating from post-capillary venules towards sites of inflammation in tissue *via* the adhesion cascade (221, 222). Upon tissue infiltration, PMNs will chemotactically migrate towards pathogens and destroy them through phagocytosis or the expulsion of DNA to form neutrophil extracellular traps (NETs) (222, 223). PMNs are also known to engage in a complex cross-talk with elements of the adaptive immune system, modulating immune effector function responses to infections (224).

PMN migratory function in response to inflammatory stimuli or environmental conditions is frequently modeled on tissue chip platforms (61, 225–227). Because of their short lifespan, PMNs are most commonly isolated from whole blood *via* density gradient separation and used immediately (Table 4). In fact, all tissue chip models utilizing PMNs described in this report thus far, have used freshly isolated PMNs. Cells obtained from donors provide high physiological relevance, however, they are subject to high degrees of biological variability and supply constraints. Cryopreserved purified human PMNs are also commercially available from biological supply vendors, however a recent study by Avci and colleagues demonstrated that PMNs exposed to a 9-week freeze-thaw cycle exhibited reduced chemotactic and polarization capability compared to freshly isolated cells when exposed to a potent PMN chemoattractant (Table 4) (233). This physiological limitation, in addition to the costs associated with purchasing frozen cells, makes isolating PMNs directly from donors the cheaper, and more common, of the two options.

**Table 4:** Leukocyte sources for tissue models.

Leukocyte	Cell Source	Main Challenges	Tissue Chip Models with TEM
Neutrophil/PMN	Blood isolation	• Biological variability • Supply constraints	• (62) • (80) • (65) • (66) • (32) • (67) • (34)
	Cryopreserved purified human PMNs	• Reduced chemotactic and polarization capability	• None
	HL-60 differentiation: Martin et al., 1990	• Diminished effector functions	• (227)
	hiPSC: Brok-Volchanskaya et al., 2019	• Decreased chemotactic response • Contains a population of immature cells lacking phagocytic activity	• None
Monocytes	Blood isolation	• Biological variability • Supply constraints	• (228) • (229)
	Immortalized monocyte-like cell lines (e.g., THP-1, U-937)	• Differing phenotypic effects and functional measures, including TEM	• (50) • (44) • (228) • (229)
	hiPSC: Yanagimachi et al., 2013; Takata et al., 2017; Cao et al., 2019; Cui et al., 2021	• Not fully characterized	• (230)
T Cells	Blood isolation	• Biological variability • Supply constraints	• (231)
	Immortalized Jurkat T cells	• Distinct actin organization and dynamics • Lack negative regulators of TCR signaling • Impaired ability to receive costimulatory signals • Large subset of differentially expressed genes	• (232)
	hiPSC: Nishimura et al., 2013; Themeli et al., 2013; Vizcardo et al., 2013; Ando et al., 2015; Maeda et al., 2016	• Optimized for cell-based immunotherapy applications only	• None

TEM, transmigration; PMN, polymorphonuclear leukocytes; hiPSC, human induced pluripotent stem cell.

Beyond acquisition from human donors, PMN-like cells can be created through differentiation protocols featuring promyelocytic cell lines, such as HL-60, or hiPSCs (Table 4) (234, 235). In one tissue chip study, PMN-like cells were produced by culturing human promyelocytic leukemia cells in complete media supplemented with 1.5% dimethyl sulfoxide (DMSO) for 4–5 days (227). This study did not include an EC layer, as the group was focused on developing a reductionist approach to study electrotaxis. Studies have demonstrated, however, that PMN-like cells derived from the HL-60 promyelocytic cell line have modified or diminished PMN effector functions, such as NET production and antimicrobial properties when compared to primary blood-derived PMNs (236, 237). As an alternative, a recent protocol produces PMNs from hiPSCs by transfection with ETV2 mRNA, a hematoendothelial programmer (238). hiPSC-derived PMNs generated by this method, however, have a decreased chemotactic response when compared to donor PMNs and contain a population of immature cells which lack phagocytic activity (238). These cells do, however, exhibit similar levels of reactive oxygen species production. To date, hiPSC-derived PMNs have not been incorporated into tissue chip models, likely due to the novelty of these protocols and the low cost of freshly isolating PMNs from blood.

### Monocytes

Monocytes are another major type of circulating leukocyte and can further differentiate into macrophages and dendritic cells (DCs). Together, these cell types play important roles in the innate immune system. Monocytes are derived from bone marrow where they originate from HSCs. Their precursors differentiate into monoblasts and then promonocytes, which finally divide into monocytes. After maturation, monocytes remain in the bone marrow for less than a day before entering circulation, where they can stay for up to 3 days before migrating into organs and tissues (239, 240). Monocytes can differentiate into macrophages and DCs, which can undergo further changes to serve more specific functions (241). For example, macrophages can polarize into pro- and anti-inflammatory phenotypes such as M1 and M2 macrophages, respectively. They are distinct from tissue-resident macrophages and DCs, which are unique to their tissue and may even have different developmental origins. The classification of monocytes, macrophages, and DCs is complex, and this diversity makes the use of monocytes and their derivatives in *in vitro* disease models complicated (240, 241).

Careful selection of monocyte source and/or differentiation method is needed for the creation of relevant *in vitro* models. Most methods identify monocytes using the monocyte/macrophage marker CD14. Similar to neutrophils, monocytes are most commonly freshly isolated from the blood, in this case selecting for peripheral blood mononuclear cells (PBMCs) (Table 4). However, they can also be obtained from commercially available immortalized monocyte-like cell lines (MCLCs), such as THP-1 and U-937 cells (Table 4). Subtle differences exist between freshly isolated and commercial monocytes. While commercial lines are well characterized and validated through gene expression and cytokine profiles, there are differences in their phenotypic effect when responding to inflammation and with functional measures such as migration (242, 243). One tissue chip study demonstrated some of these differences by comparing the responses of freshly isolated PBMCs and THP-1 cells in an inflamed liver sinusoidal organoid biochip (228). The model incorporated HUVECs and HepaRG hepatocytes, and upon stimulation with LPS, HUVECs upregulated ICAM-1 and VCAM-1. This enabled monocytes from both sources to transmigrate across the HUVEC layer, which, surprisingly, seemed to reduce the inflammatory status of the HUVECs, as measured by lower ICAM-1 and VCAM-1 levels compared to monocyte-free conditions. This is in contrast to data indicating monocyte migration further activates ECs but appears to be due to a shift to M2 polarization of macrophages upon monocyte migration. While most results were comparable between the two monocyte sources, the group did find a difference in cytokine secretion, with lower secretion by THP-1 monocytes, consistent with previous studies comparing monocyte responses to LPS. In another study, Sharifi and colleagues also used both PBMCs and THP-1 cells in their foreign body response-on-a-chip (FBROC) (229). The FBROC combined monocytes and Ti microbeads in a device separated by a vascular barrier composed of HUVECs. When monocyte chemoattractant protein-1 (MCP-1) was added to the microbead compartment, monocyte-endothelial cell interactions were activated, and THP-1 cells transmigrated into the Ti compartment. Here, they differentiated further into a pro-inflammatory M1 phenotype indicating recognition of the Ti microbeads as a foreign body. PBMCs isolated from three patient donors had varying polarization of monocytes to M1 and M2 phenotypes as indicated by different CD80/CD206 expression ratios. This highlights the potential for using PBMCs in patient-specific models of disease which is not possible with commercial MCLCs.

Monocytes derived from hiPSCs have become increasingly common, and numerous protocol exist, most of which follow a similar framework of differentiating hiPSCs to mesoderm lineage, followed by hematopoietic progenitors and finally CD14^+^ monocytes (Table 4) (244–247). These protocols have been shown to robustly produce CD14^+^ monocytes that have similar physiology to monocytes obtained from other sources. A recent study published by Ronaldson-Bouchard and colleagues utilized a commercially available monocyte differentiation kit to produce patient-specific hiPSC-derived monocytes for their multi-organ chip (230). The study demonstrated that monocytes maintained the classical CD16^−^ CD14^+^ phenotype over 4 weeks circulating in the device and were confined in the vascular channels under uninjured conditions. When cryo-injury of a heart tissue compartment was induced, monocytes were able to selectively extravasate across the vascular layer into the injured compartment while not entering the healthy compartments. As the tissue chip community continues to develop patient-specific models of disease, it is likely that hiPSC-derived monocytes will become increasingly common moving forward.

### T cells

T (**t**hymus-dependent) and B (**b**one marrow-dependent) lymphocytes contribute to the acquired, or adaptive, immune response. More specifically, this class represents the only cell types that can recognize and respond to specific antigens (248). The development of both cell types initiates from common lymphoid progenitors (CLPs), which themselves derive from multipotent HSCs. T and B lymphocyte developmental pathways share common mechanisms including the rearrangement of antigen-receptor genes, testing for successful rearrangement, and the assembly of a heterodimeric antigen receptor (249). Additionally, both T and B cell differentiation is guided by environment-specific signaling conveyed by thymic epithelial cells and bone marrow stromal cells, respectively. The established repertoire of mature T cells is long-lived and potentially self-renewing, while the repertoire of mature B cells is comprised of short-lived cells and requires replenishment from the bone marrow (250). The developmental process of lymphocytes is a complicated process, and a detailed discussion is beyond the scope of this summary but can be found elsewhere in literature (248–250). We will focus on T lymphocytes in this review, as these cells have been studied extensively using tissue chip models.

The T lymphocyte family includes two lineages distinguished by their T-cell receptor (TCR), the majority αβ TCR lineage and the minority γδ TCR lineage. The γδ lineage is less studied, and these cells are believed to be involved in processes more closely associated with the innate immune response (249). The αβ lineage undergoes further development in the thymus and is subject to developmental checkpoints known as positive and negative selection; these mechanisms ensure emerging T cells recognize self-MHC molecules yet exhibit self-tolerance (250). Additionally, the αβ lineage is comprised of sublineages distinguished by the presence of co-receptors CD4 and CD8. Functionally, these sublineages are unique as CD4 and CD8 T cells recognize and bind to MHC class II and MHC class I molecules, respectively. After recognizing specific pathogen-associated peptides in secondary lymphoid organs, T cells become activated and modify expression of adhesion molecules (251). This allows T cells to travel to sites of infection and carry out effector functions. Broadly put, cytotoxic CD8 T cells (CTLs) are involved in the direct killing of cells compromised with intracellular pathogens while effector CD4 T cells assist with the removal of extracellular pathogens and provide cytokine signaling support (250). Effector CD4 T cells can differentiate into various subtypes dependent on the local chemical environment. The T_H_1 (T Helper 1), T_H_2, and T_H_17 subtypes orchestrate the elimination of distinct pathogen classes while the T_FH_ (T follicular helper) subtype promotes B cell responses within lymph nodes. Lastly, the T_reg_ (regulatory T cell) subtype dampens the immune response and inhibits the effector functions of other subtypes (249).

The study of T lymphocytes using tissue chip models primarily focuses on their activation (252–255), trafficking (including TEM) (231, 232, 256–258) and interactions with target (usually tumor) cells (259–262). Here, we focus on interactions between T cells and ECs in the context of TEM. Very thorough reviews that cover the abovementioned topics have been written and include information relevant to other immune cells as well (208, 263–266). Traditionally, PBMCs are used as the source of T lymphocytes in tissue chips (231, 257, 258), although others have incorporated immortalized Jurkat T cells (Table 4) (232, 256). In addition, a number of approaches have been utilized for isolation of tumor infiltrating lymphocytes from human tissues utilizing enzymatic digestion, mechanical digestion, or both (267–269). Depending on the downstream application, negative selection of T cells can be utilized to prevent activation (270). Despite the fact that primary and Jurkat T cells are often used interchangeably, there are documented differences, namely, distinct actin organization and dynamics (271). Jurkat T cells lack negative regulators of TCR signaling and are impaired in their ability to receive costimulatory signals (272, 273). Additionally, a comparative analysis of post-stimulation transcription profiles revealed a substantial subset of genes was differentially expressed among primary and Jurkat T cells (274). PBMCs are isolated from blood samples using a standard density gradient centrifugation method. Beyond this, it is common to utilize an isolation kit that works on the principle of negative selection. de Haan et al. constructed an endothelium-on-a-chip model that was used to study freshly isolated T cell dynamics in response to chemokine gradients and pro-inflammatory cytokines (231). Culturing immortalized human dermal microvascular endothelial cells (HMEC-1) with stimulated T cells resulted in a near 3-fold increase of ICAM-1 expression, as well as an increased frequency of TEM relative to unstimulated cells. Interestingly, stimulated T cell TEM was mediated by a C-X-C motif chemokine ligand 12 (CXCL12) gradient but did not depend on TNF-α HMEC-1 pretreatment. It was hypothesized that perfusion of stimulated T cells established an inflammatory environment through secretion of cytokines, and this environment was not further developed through TNF-α pretreatment. Park and colleagues also aimed to study T cell-endothelium interactions and optimized a microfluidic system using stimulated Jurkat T cells (232). They reported a lack of binding between stimulated T cells and unstimulated HUVECs. This is contrary to the findings described by de Haan et al. that suggest stimulated T cells not only bind to unstimulated ECs but can subsequently extravasate. This could be due to experimental differences (rocking perfusion vs. flow, endothelium geometry, timescales examined) but may also reflect inherent features of the cell types selected (HMEC-1 vs. HUVEC and primary T cells vs. Jurkat T cells).

Protocols to generate T cells from hiPSCs have been researched extensively in the field of cancer treatment (Table 4) (275–279). These protocols are used for cell-based immunotherapy in which hiPSC methods allow for the cloning and expansion of tumor antigen-specific T cells. Essentially, tumor-specific CTLs are isolated, reprogrammed to generate hiPSCs that inherit rearranged TCR genes, expanded, and finally differentiated to yield large numbers of functionally mature CTLs (277, 279). The protocols found in literature vary but differentiation is carried out with hematopoietic/lymphopoietic cytokine cocktail treatment and co-culture with bone marrow-derived stromal cells that express the Notch ligand Delta-like 1 (OP9-DL1). Conventional methods generate a class of CTLs that is roughly 100-fold less competent compared to primary CTL counterparts, however, subtle alterations made to differentiation protocols can generate an alternative CTL class that provides comparable antigen-specific cytotoxicity relative to primary CTLs (276). Further fine tuning of protocols has resulted in enhanced hiPSC-derived CTL stability and specificity through deletion of recombination activating gene 2, thereby avoiding unwanted TCR rearrangements (280). To our knowledge hiPSC-derived T cells have not been studied in tissue chip systems, however, this endeavor would aid in the study of patient-specific T cell mechanisms as well as designing personalized treatment regimens for a broad range of diseases.

## Conclusions

The vasculature plays a central role in disease development and resolution. As tissue chip systems become more widespread for disease modeling and personalized medicine, it is important that a vascular component is included when possible. This vascular component should include relevant cell types, including endothelial cells and pericytes, for studying the impact of immune cell migration in the context of disease and inflammation. To properly target immune cell migration for therapies, careful consideration should be given to the sourcing of these cells. It is increasingly clear that mechanisms of leukocyte entry into different tissues varies, and cells from different sources can more or less faithfully recapitulate these critical phenotypes. In certain cases, “generic” endothelial cells may be sufficient, given the robustness of cell lines such as HUVECs and the ability of ECs to adopt tissue-specific phenotypes through cell-cell signaling. However, in order for personalized medicine to realize its full potential, it may be necessary to utilize tissue-specific primary cells or stem-cell derived endothelial cells, pericytes, and/or immune cells. This, of course, poses several challenges in terms of time, cost, and the general newness of many differentiation protocols. Further, generating a fully isogenic multicellular model requires careful timing of several differentiation protocols when cryopreservation is not an option. Currently, many hiPSC-derived endothelial cell methods generate immature ECs or fail to fully recapitulate the necessary immune phenotypes. This will improve with careful research and more studies characterizing tissue-specific vascular cells during inflammation. It is our hope that researchers interested in studying immune cell migration in tissue chip models will find this review helpful for identifying cell sources which express the proper leukocyte adhesion molecules for the tissue of interest.
